# The suitability of patient-reported outcome measures used to assess the impact of hypoglycaemia on quality of life in people with diabetes: a systematic review using COSMIN methods

**DOI:** 10.1007/s00125-021-05382-x

**Published:** 2021-02-02

**Authors:** Jill Carlton, Joanna Leaviss, Frans Pouwer, Christel Hendrieckx, Melanie M. Broadley, Mark Clowes, Rory J. McCrimmon, Simon R. Heller, Jane Speight

**Affiliations:** 1 School of Health and Related Research (ScHARR), University of Sheffield, Sheffield, UK; 2 Department of Psychology, University of Southern Denmark, Odense, Denmark; 3 School of Psychology, Deakin University, Geelong, VIC Australia; 4 Steno Diabetes Center Odense, Odense, Denmark; 5 The Australian Centre for Behavioural Research in Diabetes (ACBRD), Melbourne, VIC Australia; 6 School of Medicine, University of Dundee, Dundee, UK; 7 Department of Oncology and Metabolism, University of Sheffield, Sheffield, UK

**Keywords:** COSMIN, Diabetes, Hypoglycaemia, Patient-reported outcome measures, Psychometric properties, Quality of life, Questionnaire, Systematic review

## Abstract

**Aims/hypothesis:**

It is generally accepted that hypoglycaemia can negatively impact the quality of life (QoL) of people living with diabetes. However, the suitability of patient-reported outcome measures (PROMs) used to assess this impact is unclear. The aim of this systematic review was to identify PROMs used to assess the impact of hypoglycaemia on QoL and examine their quality and psychometric properties.

**Methods:**

Systematic searches (MEDLINE, EMBASE, PsycINFO, CINAHL and The Cochrane Library databases) were undertaken to identify published articles reporting on the development or validation of hypoglycaemia-specific PROMs used to assess the impact of hypoglycaemia on QoL (or domains of QoL) in adults with diabetes. A protocol was developed and registered with PROSPERO (registration no. CRD42019125153). Studies were assessed for inclusion at title/abstract stage by one reviewer. Full-text articles were scrutinised where considered relevant or potentially relevant or where doubt existed. Twenty per cent of articles were assessed by a second reviewer. PROMS were evaluated, according to COnsensus-based Standards for the selection of health Measurement INstruments (COSMIN) guidelines, and data were extracted independently by two reviewers against COSMIN criteria. Assessment of each PROM’s content validity included reviewer ratings (*N* = 16) of relevance, comprehensiveness and comprehensibility: by researchers (*n* = 6); clinicians (*n* = 6); and adults with diabetes (*n* = 4).

**Results:**

Of the 214 PROMs used to assess the impact of hypoglycaemia on QoL (or domains of QoL), eight hypoglycaemia-specific PROMS were identified and subjected to full evaluation: the Fear of Hypoglycemia 15-item scale; the Hypoglycemia Fear Survey; the Hypoglycemia Fear Survey version II; the Hypoglycemia Fear Survey-II short-form; the Hypoglycemic Attitudes and Behavior Scale; the Hypoglycemic Confidence Scale; the QoLHYPO questionnaire and the Treatment-Related Impact Measure-Non-severe Hypoglycemic Events (TRIM-HYPO) questionnaire. Content validity was rated as ‘inconsistent’, with most as ‘(very) low’ quality, while structural validity was deemed ‘unsatisfactory’ or 'indeterminate'. Other measurement properties (e.g. reliability) varied, and evidence gaps were apparent across all PROMs. None of the identified studies addressed cross-cultural validity or measurement error. Criterion validity and responsiveness were not assessed due to the lack of a ‘gold standard’ measure of the impact of hypoglycaemia on QoL against which to compare the PROMS.

**Conclusions/interpretation:**

None of the hypoglycaemia-specific PROMs identified had sufficient evidence to demonstrate satisfactory validity, reliability and responsiveness. All were limited in terms of content and structural validity, which restricts their utility for assessing the impact of hypoglycaemia on QoL in the clinic or research setting. Further research is needed to address the content validity of existing PROMs, or the development of new PROM(s), for the purpose of assessing the impact of hypoglycaemia on QoL.

**Prospero registration:**

CRD42019125153

**Graphical abstract:**

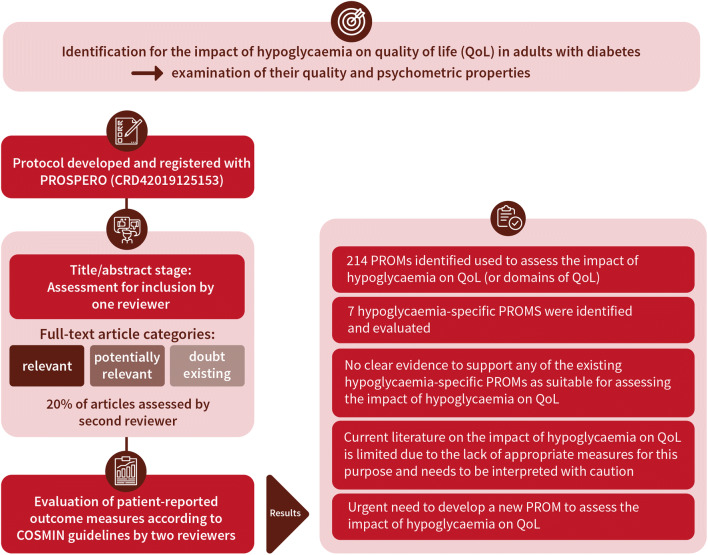

**Supplementary Information:**

The online version contains peer-reviewed but unedited supplementary material available at 10.1007/s00125-021-05382-x.


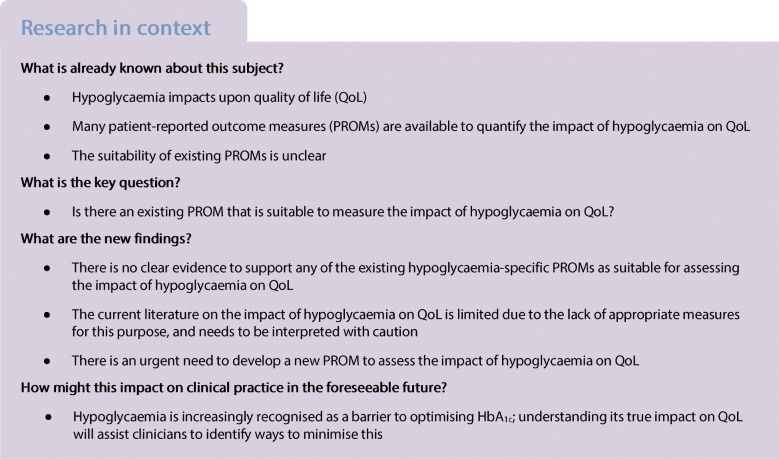


## Introduction

Both the experience and the risk of hypoglycaemia can have a serious negative impact on the quality of life (QoL) of adults with diabetes [1–8]. Living a life of quality is perhaps the ultimate goal, so protecting QoL is a daily burden for people experiencing or at risk of hypoglycaemia, and one that can be contradictory to the goals of medical therapy [8]. This may particularly be the case in those who aim for very tight glucose targets. The extent of this impact on QoL can be assessed using patient-reported outcome measures (PROMs). PROMs are questionnaires that can be used in both research and/or clinical care. PROMs complement objective data (e.g. actual blood glucose levels) by capturing the individual’s experiences in a quantifiable and standardised manner, across a range of concepts, e.g. health-related QoL, satisfaction with treatment or emotional well-being [9, 10]. When applied to the study of hypoglycaemia in diabetes, PROMs can facilitate an assessment of the psychological and economic burden of hypoglycaemia, which can be used to determine the value of therapeutic approaches to reducing hypoglycaemia frequency and severity.

Given the large number of PROMs available, it can be challenging to determine which PROM(s) to select for a given clinical or research purpose. Factors such as response burden (e.g. mode of administration, number of items [questions]), type of PROM (generic or condition-specific) and the purpose of the data collection will influence choice. However, a more fundamental issue is whether the PROM has been evaluated as ‘fit for purpose’. This evaluation should include assessment of three overall domains (validity, reliability and responsiveness), for which consensus-based standards (COnsensus-based Standards for the selection of health Measurement Instruments [COSMIN]) can be applied [11]. The COSMIN methodology and standards derive from widespread international expert consensus [11, 12] and have been applied to other PROM measures [13–17], but not yet to the assessment of the impact of hypoglycaemia on QoL.

QoL is highly subjective and has been defined in many ways and most people, intuitively, have an understanding of what it means to them [18]. Perhaps the simplest definition is that QoL is a personal evaluation of how good or bad one’s life is [19]. For the purpose of this review, and consistent with the general consensus [9], we operationalised QoL as: (1) a multidimensional construct including components such as physical well-being (e.g. pain/discomfort, mobility, fatigue), psychological well-being (e.g. mood, fear, confidence) and social well-being (e.g. stigma, participation) [20]; (2) a subjective construct based on feelings, values, experiences and priorities (therefore, we do not include objective measures, or purely functional performance or assessment instruments); and (3) a dynamic construct, which changes over time according to the person’s priorities, experiences and situation.

The objectives of this review were to: (1) identify PROMs used to assess the impact of hypoglycaemia on QoL in adults with diabetes; and (2) formally evaluate their content validity, structural validity and other measurement properties. Our intention was to provide researchers and clinicians with a robust evidence base to assist them when selecting PROMs for this purpose. The review was undertaken as part of the Hypoglycaemia REdefining SOLutions for better liVEs (Hypo-RESOLVE) project, an international collaboration of clinicians, scientists, industry partners and people with diabetes [21].

## Methods

We used the updated COSMIN guidance [12, 22–24].

### Data sources and searches

A protocol was developed and registered with PROSPERO [25]. A systematic literature search was conducted during 26–28 November 2018 to identify published evidence around the four concepts of: (diabetes) and (hypoglycaemia) and (psychosocial outcomes) and (measurement properties of measurement instruments). Databases searched include MEDLINE, EMBASE, PsycINFO, CINAHL and The Cochrane Library. Terms for psychosocial outcomes were chosen to include both generic, ‘umbrella’ terms for ‘quality and life’ and ‘well-being’ (sourced from published search filters) and specific psychosocial outcomes of diabetes known to the Hypo-RESOLVE team (e.g. fear of hypoglycaemia). In order to identify studies for the present systematic review, a validated search filter devised for retrieving studies on measurement properties of instruments in PubMed was used [26]. An example search strategy is shown in the electronic supplementary material (ESM) Methods.

### Study selection

Inclusion criteria consisted of any study design that included the primary development and/or validation of a hypoglycaemia-specific PROM used to assess the impact of hypoglycaemia on QoL in adults diagnosed with diabetes with any type, e.g. type 1, type 2 and gestational, and who have experienced hypoglycaemia. Studies of hypoglycaemia/hypoglycaemic episodes not associated with diabetes were excluded. Commentaries, reviews, opinion pieces and any other non-empirical work were also excluded. Studies were assessed for inclusion at title and abstract stage by one reviewer (JL). Full-text articles were scrutinised where considered as relevant or potentially relevant or where doubt existed. Twenty per cent of studies were assessed by a second reviewer (JC) to check for consistency. Disagreements were resolved through discussion.

### Data extraction

Data extraction included study characteristics (e.g. language; participant characteristics; recall period; analysis model), a brief summary of results and measurement properties of the PROMs. Primary outcomes included measurement properties of identified PROMs, consistent with the COSMIN checklist: PROM development; content validity; structural validity; internal consistency; cross-cultural validity/measurement invariance; reliability; measurement error; criterion validity; hypothesis testing for construct validity; and responsiveness. Definitions of the measurement properties are detailed in Table 1. In accordance with COSMIN guidelines, all data relating to PROM measurement properties were extracted independently by two reviewers (JL and JC) against the respective COSMIN criteria. Discrepancies were resolved through discussion.

**Table 1:** Definitions of measurement properties

Measurement property	Definition
Content validity	The extent to which the items in a PROM are representative of the construct they are intended to measure
Structural validity	The extent to which the items in a PROM reflect the dimensionality of the construct (i.e. the items form a single [unidimensional] scale or multiple subscales [a multidimensional scale])
Reliability: internal consistency	The extent to which there is consistency of results across items in the PROM (i.e. within a specified scale or subscale)
Reliability: test–retest	The extent to which the PROM yields scores that are reproducible (stable) over time when there has been no change in the concept being assessed
Measurement error	The systematic and random error of a person’s score on the PROM that is not attributed to changes in the construct to be measured
Criterion validity	The extent to which the scores of a PROM reflect the scores of a test or measure considered to be the ‘gold standard’
Hypothesis testing for construct validity	The extent to which the scores of a PROM are consistent with hypotheses. For example, with regard to internal relationships, relationships to scores of other instruments or differences between relevant groups. It is based on the assumption that the PROM is a valid measure of the construct
Responsiveness	The ability of a PROM to detect change, as expected, over time in the construct to be measured when there is a true change in a person’s condition or treatment
Cross-cultural validity	The extent to which the measurement properties of the translated or culturally adapted PROM reflect the performance of the original version of the PROM

### Content validity assessment

**Fig. 1:**
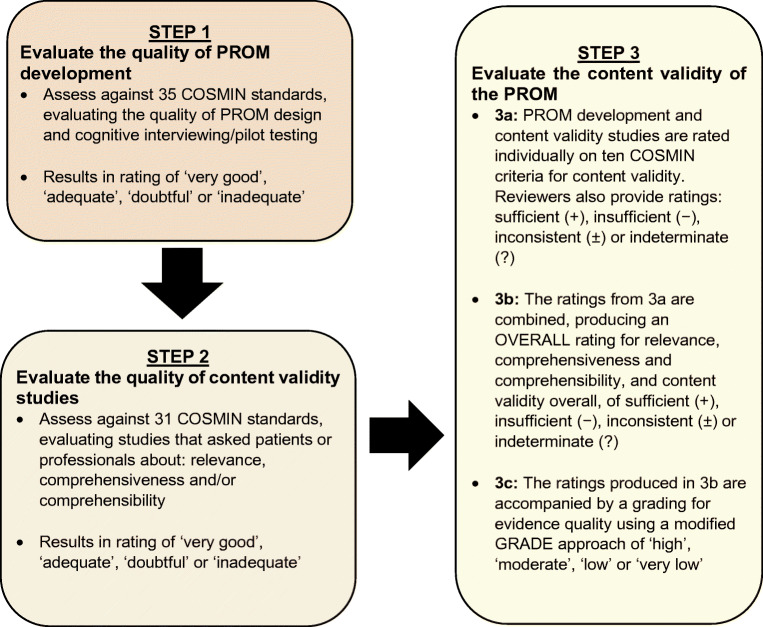
COSMIN assessment of content validity

Content validity is the extent to which a PROM is deemed to reflect the construct of interest and, arguably, the most fundamental aspect of scale selection [27]. The methodological quality of the PROM development studies and other studies supplementing content validity were assessed using COSMIN standards [28]. The assessment involves three steps (see Fig. 1): (1) evaluation of the quality of the PROM development; (2) evaluation of the quality of any additional content validity studies on the PROM (if available); and (3) evaluation of the content validity of the PROM based on the quality and results of the available studies and the PROM itself. Steps 1 and 2 result in a rating of each COSMIN standard ranked on a four-point scale: ‘very good’, ‘adequate’, ‘doubtful’ and ‘inadequate’. Total ratings are then determined using the lowest rating for any item for that study (i.e. worst score counts) [22].

Step 3 consists of three sub-stages. Step 3a incorporates reviewer ratings of the identified PROMs whereby reviewers consider relevance, comprehensiveness and comprehensibility. We sought ratings from three key stakeholder groups: (1) researchers (including those with expertise in systematic reviewing, QoL research and psychological aspects of diabetes) (*n* = 6); (2) clinicians (*n* = 6); and (3) adults with diabetes (*n* = 4), including two representatives of the Hypo-RESOLVE Patient Advisory Committee (PAC). All reviewers provided independent ratings of the PROMs based on several criteria: (1) the construct of interest (i.e. does the PROM include items that are relevant in measuring the impact of hypoglycaemia on QoL?); (2) the population of interest; (3) the context of use of interest (i.e. is the PROM suitable for use in research and/or clinical practice?); (4) the appropriateness of response options; (5) the appropriateness of the recall period; (6) the comprehensiveness (i.e. does the PROM assess the impact of hypoglycaemia on QoL as a whole, or only on select domains of QoL?); (7) the suitability/clarity of the PROM instructions; (8) whether PROM items and response options are understandable; (9) the appropriateness of PROM item wording; and (10) the extent to which response options are appropriate to the question being asked. A majority rating was determined for each group (researcher, clinician and PAC). The group ratings were then consolidated to produce an overall reviewer rating for each PROM. Table 2 details how relevance, comprehensiveness and comprehensibility were assessed.

**Table 2:** COSMIN criteria and rating system for evaluating the content validity of the PROMs (adapted from Terwee et al [28]), with an example shown in italics

Name of PROM (or subscale)	PROM development study (+/−/±/?)	Content validity study 1 (+/−/±/?)	Content validity study 2^a^ (+/−/±/?)	Rating of reviewers (+/−/±/?)	Overall ratings per PROM (+/−/±/?)	Quality of evidence (High, moderate, low, very low)
*The ABC-QoL*	*Jones et al, 2015*	*Smith et al, 2016*				
Relevance						
1. Are the included items relevant for the construct of interest?^b^	+	+		+		
2. Are the included items relevant for the target population of interest?^c^	+	+		+		
3. Are the included items relevant for the context of use and interest?^d^	−	−		−		
4. Are the response options appropriate?	+	−		+		
5. Is the recall period appropriate?	+	+		+		
RELEVANCE RATING	+	+		+	+	
Comprehensiveness						
6. Are all key concepts covered?	−	−		−		
COMPREHENSIVENESS RATING	−	−		−	−	
Comprehensibility						
7. Are the PROM instructions understood by the population of interest as intended?	+	+				
8. Are the PROM items and response options understood by the population of interest as intended?	+	+				
9. Are the PROM items appropriately worded?				+		
10. Do the response options match the question?				+		
COMPREHENSIBILITY RATING	±	±		±	±	
CONTENT VALIDITY RATING					±	High

+, −, ±, and ? denote sufficient, insufficient, inconsistent, indeterminate

^a^More columns to be added if more content validity studies are available

^b^For this review, the construct of interest was ‘impact of hypoglycaemia on QoL’

^c^For this review, the population was ‘adults with diabetes’

^d^For this review, the context of interest was ‘research use in a clinical and/or research setting’

Step 3b involves summarising the results of all available studies to provide an overall rating of relevance, comprehensiveness and comprehensibility and an overall content validity rating. This results in an outcome of ‘sufficient’, ‘insufficient’, ‘inconsistent’ or ‘indeterminate’. Finally, in Step 3c, the overall ratings determined in Step 3b are accompanied by a grading of the quality of the evidence using a modified Grading of Recommendations Assessment, Development and Evaluation (GRADE) approach [29]. Using the modified GRADE approach, the quality of evidence is graded as ‘high’, ‘moderate’, ‘low’ or ‘very low’. The GRADE approach uses five factors to consider the quality of the evidence: risk of bias, inconsistency, indirectness, imprecision and publication bias [29]. Detailed information of the rating process is reported elsewhere [28]. The resultant evaluation of content validity includes an overall rating of: + (‘satisfactory’); − (‘unsatisfactory’); ± (‘inconsistent’); or ? (‘indeterminate’), with a measure of the quality of the evidence to support the content validity rating (‘high’, ‘moderate’, ‘low’, ‘very low’). A worked example of content validity rating and scoring is shown in Table 2. Detailed information on the COSMIN methodology applied is reported elsewhere [28].

### Assessment of other psychometric properties

Table 1 defines each of the psychometric properties assessed. As above, a COSMIN rating was determined by assessment across the criteria for measurement properties using the same rating scale (‘sufficient’, ‘insufficient’, ‘inconsistent’ or ‘indeterminate’). The assessment of the quality of the evidence was applied using the GRADE approach. This results in a rating of: + (‘satisfactory’); − (‘unsatisfactory’); ± (‘inconsistent’); or ? (‘indeterminate’), with a measure of the quality of the evidence to support the structural validity rating (‘high’, ‘moderate’, ‘low’, ‘very low’). Full information on the COSMIN methodology applied in this review is reported elsewhere [23].

### Quality assurance of the review

The quality of this review was assessed against a COSMIN checklist that was designed to evaluate the quality of systematic reviews of PROMs [30] (ESM Table 1).

## Results

**Fig. 2:**
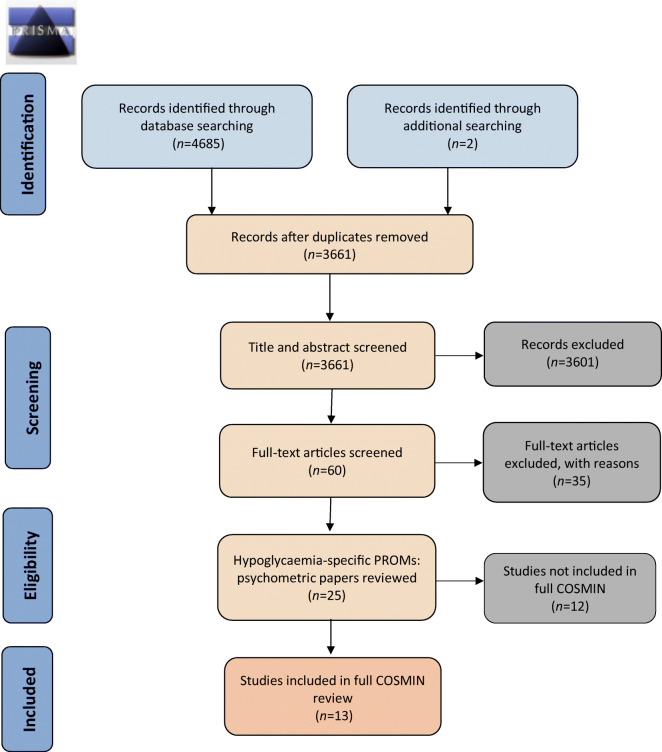
PRISMA 2009 Flow Diagram: hypoglycaemia-specific PROMs used to assess the impact of hypoglycaemia on QoL

The search returned a total of 3661 unique records, from which 214 PROMs were identified as used in studies to assess the impact of hypoglycaemia on QoL or subdomains of QoL (Fig. 2, Table 3). Of these, 17 PROMs were initially identified as hypoglycaemia-specific and for consideration in this review, and nine were subsequently excluded following further scrutiny of the instruments. PROMs were excluded if they were: hypoglycaemia symptom measures that assessed attitudes, awareness and/or attitudes to awareness of symptoms (*n* = 3); related to specific treatments (*n* = 2); only a subscale of an overall PROM (*n* = 2); or not available for full inspection (*n* = 2). Consequently, the current review includes eight hypoglycaemia-specific PROMs that have been used to assess the impact of hypoglycaemia on QoL or at least one aspect of QoL: the Fear of Hypoglycemia 15-item scale (FH-15); the Hypoglycemia Fear Survey (HFS); the Hypoglycemia Fear Survey version II (HFS-II); the HFS-II short-form; the Hypoglycemic Attitudes and Behavior Scale (HABS); the Hypoglycemic Confidence Scale (HCS); the QoLHYPO questionnaire and the Treatment-Related Impact Measure-Non-severe Hypoglycemic Events (TRIM-HYPO) (Table 4).

**Table 3:** Type and number of PROMs identified in title and abstract sift

Type of PROM measure	Number of PROMs
Designed for completion by children/adolescents	22
Designed for completion by adults	192
Generic	82
Diabetes-specific	51
Treatment-specific	37
Glucose-monitoring-specific	5
Hypoglycaemia-specific	17
Total	214

**Table 4:** PROMs identified that have been used to assess the impact of hypoglycaemia on QoL (or its subdomains) in people with diabetes

PROM	Recall period	*N* domains (items)	Domains assessed by PROM (*n* items)	Response options	Total score range	Origin	Validated English version available for review
FH-15	Not stated	3 (15)	Fear (7), avoidance (3), interference (5)	Never, almost never, sometimes, almost always, every day. 1–5 scale	15–75	Spain	No—Spanish only version
HFS	Not stated	2 (27)	Behaviour (10), worry (17)	Never, rarely, sometimes, often, very often. 1–5 scale	27–135	USA	Yes
HFS-II	6 months	2 (33)	Behaviour (15), worry (18)	Never, rarely, sometimes, often, almost always. 0–4 scale	0–132	USA	Yes
HFS-II short-form	6 months	2 (11)	Behaviour (5), worry (6)	Never, rarely, sometimes, often, almost always. 0–4 scale	0–44	USA	Yes
HABS	Present	3 (14)	Avoidance (4), confidence (5), anxiety (5)	Strongly disagree, disagree, neutral, agree, strongly agree. 1–5 scale	14–70	USA	Yes
HCS	Not stated	1 (9)	Confidence (9)	Not confident at all, a little confident, moderately confident, very confident. 1–4 scale	9–36	USA	Yes
QoLHYPO	Not stated	Not stated (13)	Not reported	Never, rarely, sometimes, often, always. 0–4 scale	0–52	Spain	No—Spanish only version
TRIM-HYPO	Past 30 days	5 (33)	Daily function (7), Emotional wellbeing (7), Work productivity (9), Sleep disruption (5), Diabetes management (5)	Varies per item. 1–5 scale	0–100	France, Germany, UK, USA	Yes

### Overall COSMIN assessment of PROMs

The overall results of the COSMIN assessment are shown in Table 5. There are considerable evidence gaps for the measurement properties of most of the PROMs. The HFS-II, QoLHYPO and TRIM-HYPO were the only instruments that could be rated across all the measurement properties.

**Table 5:** Summary of psychometric properties of hypoglycaemia-specific PROMs used to assess the impact of hypoglycaemia on QoL

PROM	Content validity		Structural validity		Reliability: internal consistency		Reliability: test–retest		Hypothesis testing for construct validity	
	Rating of results	Quality of evidence	Rating of results	Quality of evidence	Rating of results	Quality of evidence	Rating of results	Quality of evidence	Rating of results	Quality of evidence
FH-15	±	Low	−	Moderate	+	Moderate	NR	NR	?	Moderate
HFS	±	Moderate	−	Moderate	?	Moderate	−	Very low	NR	NR
HFS-II	±	Low	−	Moderate	+	Moderate	−	Moderate	?	Moderate
HFS-Norwegian	NR	NR	−	High	+	High	+	High	NR	NR
HFS-Singapore	NR	NR	−	High	+	High	?	High	?	High
HFS-Spanish	NR	NR	−	Moderate	?	Moderate	?	Very low	?	Moderate
HFS-Swedish	NR	NR	−	Moderate	+	Moderate	NR	NR	?	Moderate
HFS-II short-form	NR	NR	−	High	+	High	NR	NR	?	High
HABS	±	Very low	−	Moderate	+	Moderate	NR	NR	?	Moderate
HCS	±	Low	−	Moderate	+	Moderate	NR	NR	?	Moderate
QoLHYPO	±	Moderate	−	Moderate	+	Moderate	+	Moderate	?	Moderate
TRIM-HYPO	±	Low	?	Moderate	±	Moderate	−	Very low	±	Moderate

±, inconsistent results; −, unsatisfactory results; +, satisfactory results; NR, not reported; ?, indeterminate

### Content validity

ESM Table 2 summarises the key characteristics and COSMIN quality assessment of the PROM development studies. For five of the seven PROMs, there was evidence that adults with diabetes were involved in item generation (HFS, HABS, HCS, QoLHYPO and TRIM-HYPO). COSMIN quality ratings ranged from ‘inadequate’ (HFS, HABS and QoLHYPO), to ‘doubtful’ (HFS-II, HCS and TRIM-HYPO), to ‘very good’ (FH-15). The developers of the HFS-II short-form do not report on content validity, due to the scale being developed based on existing items in the HFS-II [31].

ESM Table 3 details characteristics of the PROM development studies. The overall quality of the PROM development studies was classified as ‘very good’ (FH-15), ‘inadequate’ (HFS, HABS and QoLHYPO) or ‘doubtful’ (HFS-II, HCS and TRIM-HYPO). Only five of the PROMs provided evidence of concept elicitation (all of which were of ‘doubtful’ or ‘inadequate’ quality) (HFS, HABS, HCS, QoLHYPO and TRIM-HYPO). The COSMIN rating for the PROM design ranged from ‘inadequate’ (HFS, HABS and QoLHYPO), to ‘doubtful’ (HFS-II, HCS and TRIM-HYPO), to ‘very good’ (FH-15). Three of the PROMs (HFS, QoLHYPO and TRIM-HYPO) reported on content validity. During the development of the HFS, health professionals were asked about the relevance and comprehensiveness of the PROM (‘doubtful’ COSMIN quality rating) [32]. For the QoLHYPO, adults with diabetes were asked about the comprehensibility, but not relevance, of the PROM (‘doubtful’ COSMIN quality rating) [33]. During the development of the TRIM-HYPO, adults with diabetes were asked about the comprehensibility and relevance of the PROM, but were not asked about comprehensiveness of the PROM ('doubtful' COSMIN quality rating) [34]. Aside from the development studies, no further studies were identified that independently assessed the content validity of the PROMs.

ESM Table 4 details the consensus ratings for the three groups of reviewers (researchers, clinicians, people living with diabetes), and an overall reviewer consensus rating for each PROM. FH-15 had an overall reviewer rating of ‘sufficient’; HFS-II, HABS, HCS and TRIM-HYPO were rated as ‘inconsistent’. For two of the PROMs (HFS and QoLHYPO), relevance, comprehensiveness and comprehensibility ratings resulted in a combination whereby COSMIN guidance is not explicit, and, thus, an overall rating could not be applied [28].

### Structural validity

Twelve studies assessed the structural validity of the PROMs, all of which were reported in the development papers (ESM Table 5). No independent assessments of the structural validity were identified. Four studies examined the structural validity of a cultural adaptation/language translation of the HFS [35–38]. A further study assessed the structural validity of the short-form of HFS-II [31]. COSMIN quality ratings of the HFS-Norwegian, HFS-Singapore and HFS short-form were ‘very good’ and ratings were ‘adequate’ for the remaining PROMs. The same principles as noted above were applied to assess the quality of the evidence for these instruments. The quality of evidence for the HFS-Norwegian, HFS-Singapore and HFS-II short-form instruments was assessed as ‘high’*.* The HFS-Spanish, HFS-Swedish and TRIM-HYPO instruments were assessed as ‘moderate’. Many of the studies reported exploratory factor analysis (EFA) (rather than the confirmatory factor analysis required to receive a ‘satisfactory’ rating). Those studies reporting confirmatory factor analysis (language versions of the HFS) did so to examine whether the expected two-factor structure (observed for the original HFS) fitted their dataset. However, they all rejected this a priori-defined structure, and therefore went on to explore the latent structure of the tool using EFA.

### Internal consistency reliability

Thirteen studies were identified that reported evidence of the internal consistency of the PROMs [31–34, 36–43]. Some were undertaken by the instrument developers and some were independent assessments (ESM Table 6). Most studies [32, 33, 36, 39, 40, 42, 43] had an ‘adequate’ COSMIN quality rating. Five studies had a ‘very good’ COSMIN quality rating [31, 34, 37, 38, 41].

### Reliability (test–retest)

Seven studies were identified that assessed the test–retest reliability of a PROM measure. Four of the studies were conducted by the instrument developers (FH-15, HFS-II, QoLHYPO and TRIM-HYPO). The remaining studies were assessments of the language versions of the HFS instrument (ESM Table 7). Four studies had an ‘adequate’ COSMIN quality rating [32, 33, 36, 40]. Two studies had a ‘very good’ COSMIN quality rating [37, 38]. One study had a 'doubtful' COSMIN quality rating [34].

### Hypothesis testing for construct validity

Ten studies reported on hypothesis testing for construct validity (ESM Table 8) [31, 33–36, 38–40, 42, 43]. Of these, nine were comparing with other outcome measurement instruments (convergent validity) [31, 33–36, 38, 40, 42, 43]. These were HFS-II, HFS-Spanish, HFS-Singapore, HFS-Sweden, HFS-II short-form, HABS, HCS, QoLHYPO and TRIM-HYPO. Six studies included comparisons between subgroups (discriminative or known-groups validity) [34, 38–40, 42, 43]. These were FH-15, HFS-II, HFS-Singapore, HABS, HCS and TRIM-HYPO instruments.

### Other psychometric properties

No studies were found to demonstrate evidence for cross-cultural validity, measurement error, criterion validity or responsiveness.

## Discussion

This systematic review has summarised and critically evaluated published evidence on the psychometric characteristics of PROMs used to assess the impact of hypoglycaemia on QoL in adults with diabetes using COSMIN methodology. Our intention was to provide an evidence base that would help researchers and clinicians when selecting PROMs, based on the robust and comprehensive consensus-based COSMIN criteria. We identified eight PROMs that had been developed to assess the subjective impact of hypoglycaemia on QoL or a subdomain of QoL.

None of the PROMs included in this review had a ‘high’ rating for content validity (in relation to assessing the impact of hypoglycaemia on QoL), which is arguably the most important measurement property of a PROM [28, 44]. All had ‘inconsistent’ COSMIN ratings for content validity, but the quality of the evidence to support those ratings was greater for the HFS and QoLHYPO. To that end, there is some support to recommend the use of HFS and QoLHYPO instruments in research studies and/or clinical practice. However, it is important to acknowledge the conceptual framework from which these two instruments were developed, and how this diverges from our operationalisation of the concept of QoL (i.e. multidimensional, subjective and changing over time). The HFS was developed to measure fear of hypoglycaemia through two subscales—behaviour and worry. Fear is arguably a very specific aspect of the psychological subdomain of QoL. Furthermore, the developers were not explicit in describing the target population for the instrument (i.e. their sample included people with ‘insulin-dependent’ diabetes, but it is unclear whether this included people type 1 and/or type 2 diabetes, and whether it is also applicable to people who manage their diabetes without insulin but experience hypoglycaemia). While the content of the QoLHYPO instrument includes items that assess various domains of QoL (e.g. social relationships, mood, daily activities), it was designed for use only by people with type 2 diabetes. Furthermore, there have been no translations beyond the original Spanish version. Consequently, the format and layout of the QoLHYPO is not clear for English-speaking researchers, and the developers provide no information on domains. Further investigation would be required to determine the suitability of the QoLHYPO instrument in measuring the impact of hypoglycaemia in people with type 1 diabetes and in other language groups.

We have included details of psychometric properties of the PROMs identified as part of the original literature search. However, it is plausible that additional papers have also reported psychometric properties for one or more of the included PROMs (particularly in intervention studies). To that end, the information on measurement properties reported here should not be considered exhaustive. We did not adopt the approach taken by (some of) the PROM authors to consider HbA_1c_ as the ‘gold standard’ in the assessment of criterion validity and criterion approach to responsiveness. Studies have shown that HbA_1c_ it is not a reliable indicator of whether an individual experiences hypoglycaemia [45, 46], nor a surrogate for QoL [47], nor of the impact or burden of hypoglycaemia. Advances in glucose monitoring technologies are continually changing our understanding of diabetes and are contributing to a better understanding of the lived experience of diabetes and hypoglycaemia. Consequently, it may be appropriate in future studies to consider ‘time in range’ or ‘time in hypoglycaemia’ as a marker for the impact of hypoglycaemia on QoL—but the extent to which this will reflect the subjective experience has yet to be elucidated. In the absence of an agreed ‘gold standard’, it is not possible to determine the assessment of any criterion validity or criterion approach to responsiveness for any PROM.

In this systematic review, we followed the robust and comprehensive guidance developed by the COSMIN initiative [23, 28]. However, it is not without its limitations. The assessment of content validity and psychometric performance of PROMs is determined by taking the lowest rating of any standard in the criteria (i.e. the ‘worst score counts’ principle) [22, 28]. This means that a study could be rated as ‘very good’ or ‘good’ on all but one criterion; however, the overall rating could be affected by a ‘doubtful’ or ‘inadequate’ rating, thus reducing the overall score to ‘doubtful’ (or ‘inadequate’). The omission of one key component in reporting (such as whether interviews were recorded and transcribed verbatim) can result in a lower overall content validity rating, which could be argued as overly harsh and should be recognised as a limitation of the COSMIN approach. Where appropriate within this review, we consistently rated in favour of the PROM (rather than assuming the worst). Another limitation of the COSMIN approach was identified in the guidance for determining content validity ratings of studies. Here we noted that there was no information on how to determine overall content validity rating with the combinations achieved. We have documented our approach; however, if the review was to be replicated, others may opt to ‘down-grade’ the overall content validity rating. Furthermore, as part of the content validity assessment, we sought to include the opinion of stakeholders. The COSMIN guidance does not advise on how to ratify ratings should there be conflicting opinions between or within stakeholder groups.

It should be noted that some of the PROMs included within this review are legacy or ‘first generation’ measures; that is, they were developed at a time when there were no international standards for instrument development methods, so these were either not reported, or reported selectively or in little detail. Similarly, the way in which PROMs are developed has changed over time [27]. It is now more common to report the methodological steps undertaken during the instrument development phase. The COSMIN ratings should therefore be interpreted with a degree of caution, and do not provide evidence that the instrument development was not rigorous or that the instruments are not ‘fit for purpose’, but rather expose an absence of key evidence.

While there is published evidence of studies that report hypoglycaemia to negatively impact upon QoL [1–8], we have identified that those that utilise hypoglycaemia-specific PROMs have inadequate reliability and validity for this specific purpose. Thus, the current literature on the impact of hypoglycaemia on QoL is limited (if not flawed) and needs to be interpreted with caution. Given that the content validity of the instruments was lacking, it is plausible that hypoglycaemia impacts individuals in ways that are currently not being measured. It may be that the items within the instruments are no longer relevant (e.g. due to changes in diabetes treatments, monitoring, society, language use), or that the items are not comprehensive enough to fully capture the ways in which hypoglycaemia affects adults in the modern world.

In conclusion, none of the PROMs identified had sufficient evidence to demonstrate satisfactory content validity, i.e. they do not assess the impact of hypoglycaemia on QoL in adults living with diabetes. Furthermore, most were also limited in their published evidence of reliability, validity and responsiveness. There is an urgent need to follow contemporary guidance [27, 48–50] to develop new instruments that can assess the impact of hypoglycaemia on QoL.

## Supplementary Information

ESM (PDF 281 KB)

## Data Availability

Data are available on request from the authors.

## Abbreviations

COSMIN: COnsensus-based Standards for the selection of health Measurement INstruments
EFA: Exploratory factor analysis
FH-15: Fear of Hypoglycemia 15-item scale
GRADE: Grading of Recommendations Assessment, Development and Evaluation
HABS: Hypoglycemic Attitudes and Behavior Scale
HCS: Hypoglycemic Confidence Scale
HFS: Hypoglycemia Fear Survey
HFS-II: Hypoglycemia Fear Survey version II
Hypo-RESOLVE: Hypoglycaemia REdefining SOLutions for better liVEs
PAC: Patient Advisory Committee
NSHE: Non-severe hypoglycaemic event
PROM: Patient-reported outcome measure
QoL: Quality of life
QoLHYPO: QoLHYPO questionnaire
TRIM-HYPO: Treatment-Related Impact Measure-Non-severe Hypoglycemic Events

## References

[1] Hendrieckx C, Ivory N, Singh H, Frier BM, Speight J (2019). Impact of severe hypoglycaemia on psychological outcomes in adults with type 2 diabetes: a systematic review. Diabet Med.

[2] Wieringa TH, de Wit M, Twisk JWR, Snoek FJ (2018). Does hypoglycaemia affect the improvement in QoL after the transition to insulin in people with type 2 diabetes?. J Endocrinol Investig.

[3] LaManna J, Litchman ML, Dickinson JK (2019). Diabetes education impact on hypoglycemia outcomes: a systematic review of evidence and gaps in the literature. Diabetes Educ.

[4] Barendse S, Singh H, Frier BM, Speight J (2012). The impact of hypoglycaemia on quality of life and related patient-reported outcomes in type 2 diabetes: a narrative review. Diabet Med.

[5] Lundkvist J, Berne C, Bolinder B, Jönsson L (2005). The economic and quality of life impact of hypoglycaemia. Eur J Health Econ.

[6] Jacobson AM, Braffett MS, Cleary PA, Gubitosi-Klug RA, Larkin ME, the DCCT/EDIC Research Group (2013). A 23-year follow-up of the diabetes control and complications/epidemiology of diabetes interventions and complications cohort. Diabetes Care.

[7] Rossi MC, Nicolucci A, Ozzello A (2019). Impact of severe and symptomatic hypoglycemia on quality of life and fear of hypoglycemia in type 1 and type 2 diabetes: results of the Hypos-1 Observational Study. Nutr Metab Cardiovasc Dis.

[8] Heindrieckx C, Gonder-Frederick L, Heller SR, Snoek FJ, Speight J (2020). How has psycho-behavioural research advanced our understanding of hypoglycaemia in type 1 diabetes?. Diabet Med.

[9] Speight J, Reaney MD, Barnard KD (2009). Not all roads lead to Rome—a review of quality of life measurement in adults with diabetes. Diabet Med.

[10] Reaney M, Gwaltney C (2014). Measuring and interpreting patient-reported outcome data from clinical trials of diabetes medication. J Diabetes Res Clin Metab.

[11] Mokkink LB, Terwee CB, Patrick DL (2010). The COSMIN study reached international consensus on taxonomy, terminology, and definitions of measurement properties for health-related patient-reported outcomes. J Clin Epidemiol.

[12] Terwee CB, Prinsen CAC, Chiarotto A (2018). COSMIN methodology for evaluating the content validity of patient-reported outcome measures: a Delphi study. Qual Life Res.

[13] Chiarotto A, Ostelo RW, Boers M, Terwee CB (2018). A systematic review highlights the need to investigate the content validity of patient-reported outcome measures for physical functioning in patients with low back pain. J Clin Epidemiol.

[14] Craxford S, Deacon C, Myint Y, Ollivere B (2019). Assessing outcome measures used after rib fracture: a COSMIN systematic review. Injury.

[15] Rezai M, Kolne K, Bui S, Lindsay S (2020). Measures of workplace inclusion: a systematic review using the COSMIN methodology. J Occup Rehabil.

[16] Ortega-Avila AB, Cervera-Garvi P, Ramos-Petersen L, Chicharro-Luna E, Gijon-Nogueron G (2019). Patient-reported outcome measures for patients with diabetes mellitus associated with foot and ankle pathologies: a systematic review. J Clin Med.

[17] Powell PA, Carlton J, Buckley Woods H, Mazzone P (2020). Measuring quality of life in Duchenne muscular dystrophy: a systematic review of the content and structural validity of commonly used instruments. Health Qual Life Outcomes.

[18] Speight J, Holmes-Truscott E, Hendrieckx C, Skovlund S, Cooke D (2020). Assessing the impact of diabetes on quality of life: what have the past 25 years taught us?. Diabet Med.

[19] McGee HM, O’Boyle CA, Hickey A, O’Malley K, Joyce CR (1991) Assessing the quality of life of the individual: The SEIQoL with a healthy and a gastroenterology unit population. Psychol Med 21(3):749–759 10.1017/s0033291700022388 1946863

[20] Schipper HC, Clinch JJ, Olweny CLM, Spilker B (1996). Quality of life studies: definitions and conceptual issues. Quality of life and pharmacoeconomics in clinical trials.

[21] de Galan BE, McCrimmon RJ, Ibberson M, for the Hypo-RESOLVE Consortium (2020). Reducing the burden of hypoglycaemia in people with diabetes through increased understanding: design of the Hypoglycaemia REdefining SOLutions for better liVEs (Hypo-RESOLVE) project. Diabet Med.

[22] Terwee CB, Mokkink LB, Knol DL, Ostelo RW, Bouter LM, de Vet HC (2012). Rating the methodological quality in systematic reviews of studies on measurement properties: a scoring system for the COSMIN checklist. Qual Life Res.

[23] Mokkink L, Prinsen C, Patrick D et al (2018) COSMIN methodology for systematic reviews of patient-reported outcome measures (PROMs)—user manual. Available from https://www.cosmin.nl/wp-content/uploads/COSMIN-syst-review-for-PROMs-manual_version-1_feb-2018.pdf. Accessed 1 Dec 2018

[24] Prinsen CAC, Mokkink LB, Bouter LM (2018). COSMIN guideline for systematic reviews of patient-reported outcome measures. Qual Life Res.

[25] Carlton J, Leaviss J, Clowes M, Hendrieckx C, Pouwer F, Speight J (2020) Psychometric characteristics of patient-reported outcome measures of the impact of hypoglycaemia on quality of life (QoL) in people with diabetes (PwD): a systematic review using COSMIN. PROSPERO 2019 CRD42019125153 Available from https://www.crd.york.ac.uk/prospero/display_record.php?ID=CRD42019125153. Registered 27 Feb 2020

[26] Terwee CB, Jansma EP, Riphagen II, de Vet HCW (2009). Development of a methodological PubMed search filter for finding studies on measurement properties of measurement instruments. Qual Life Res.

[27] U.S Department of Health and Human Services Food and Drug Administration (FDA) (2009) Guidance for industry: patient-reported outcome measures: use in medicinal product development to support labelling claims. Available from https://www.fda.gov/media/77832/download. Accessed 1 Dec 2018

[28] Terwee CB, Prinsen CAC, Chiarotto A et al (2018) COSMIN methodology for assessing the content validity of PROMs: user manual. Available from https://www.cosmin.nl/wp-content/uploads/COSMIN-methodology-for-content-validity-user-manual-v1.pdf. Accessed 1 Dec 2018

[29] Guyatt GH, Oxman AD, Vist GE (2008). GRADE: an emerging consensus on rating quality of evidence and strength of recommendations. BMJ.

[30] Terwee CB, Prinsen CAC, Ricci Garotti MG, Suman A, de Vet HCW, Mokkink LB (2016). The quality of systematic reviews of health-related outcome measurement instruments. Qual Life Res.

[31] Grabman J, Vajda Bailey K, Schmidt K (2017). An empirically derived short form of the Hypoglycaemia Fear Survey II. Diabet Med.

[32] Cox DJ, Irvine A, Gonder-Frederick L, Nowacek G, Butterfield J (1987). Fear of hypoglycemia: quantification, validation, and utilization. Diabetes Care.

[33] Orozco-Beltràn D, Artola S, Jansa M, Lopez de la Torre-Casares M, Fuster E (2018). Impact of hypoglycemic episodes on health-related quality of life of type-2 diabetes mellitus patients: development and validation of a specific QoLHYPO© questionnaire. Health Qual Life Outcomes.

[34] Brod M, Højbjerre L, Bushnell DM, Hansen CT (2015) Assessing the impact of non-severe hypoglycemic events and treatment in adults: development of the Treatment-Related Impact Measure-Non-severe Hypoglycemic Events (TRIM-HYPO). Qual Life Res 24(12):2971–2984. 10.1007/s11136-015-1023-6 10.1007/s11136-015-1023-6 26094008

[35] Anderbro T, Amsberg S, Wredling R et al (2008) Psychometric evaluation of the Swedish version of the Hypoglycaemia Fear Survey. Patient Educ Couns 73(1):127–131. 10.1016/j.pec.2008.03.022 10.1016/j.pec.2008.03.022 18472383

[36] Tasende C, Rubio JA, Alvarez J (2018). Spanish translation, adaptation and validation of the Hypoglycemia Fear Survey in adults with type 1 diabetes in the Community of Madrid. Endocrinol Diabetes Nutr.

[37] Graue M, Iversen MM, Wentzel-Larsen T, Rokne B, Haugstvedt A (2013). Assessing fear of hypoglycemia among adults with type 1 diabetes - psychometric properties of the Norwegian version of the Hypoglycemia Fear Survey II questionnaire. Norsk Epidemiologi.

[38] Lam AYR, Xin X, Tan WB, Gardner DSL, Goh SY (2017). Psychometric validation of the Hypoglycemia Fear Survey-II (HFS-II) in Singapore. BMJ Open Diabetes Res Care.

[39] Anarte Ortiz MT, Caballero FF, Ruiz de Adana MS (2011). Development of a new fear of hypoglycemia scale: FH-15. Psychol Assess.

[40] Gonder-Frederick LA, Schmidt KM, Vajda KA (2011). Psychometric properties of the Hypoglycemia Fear Survey-II for adults with type 1 diabetes. Diabetes Care.

[41] Pinhas-Hamiel O, Tisch E, Levek N (2017). Sexual lifestyle among young adults with type 1 diabetes. Diabetes Metab Res Rev.

[42] Polonsky WH, Fisher L, Hessler D, Edelman SV (2015). Development of a new measure for assessing insulin delivery device satisfaction in patients with type 1 and type 2 diabetes. Diabetes Technol Ther.

[43] Polonsky WH, Fisher L, Hessler D, Edelman SV (2017). Investigating hypoglycemic confidence in type 1 and type 2 diabetes. Diabetes Technol Ther.

[44] Terwee CP, Prinsen CAC, Chiarotto A (2018). COSMIN standards and criteria for evaluating the content validity of health-related patient-reported outcome measures: a Delphi study. Qual Life Res.

[45] Hendrieckx C, Halliday JA, Bowden JP (2014). Severe hypoglycaemia and its association with psychological well-being in Australian adults with type 1 diabetes attending specialist tertiary clinics. Diabetes Res Clin Pract.

[46] Miller KM, Foster NC, Beck RW, T1D Exchange Clinic Network (2015). Current state of type 1 diabetes treatment in the U.S.: updated data from the T1D Exchange clinic registry. Diabetes Care.

[47] Walker J, Bradley C (2002). Assessing the quality of life of adolescents with diabetes: using the SEIQoL, DQoL, patient and diabetes specialist nurse ratings. Pract Diab Int.

[48] Wild D, Grove A, Martin M (2005). Principles of good practice for the translation and cultural adaptation process for patient-reported outcomes (PRO) measures: Report of the ISPOR task force for translation and cultural adaptation. Value Health.

[49] International Consortium for Health Outcomes Measurement. Measuring results that matter: type 1 and type 2 diabetes in adults data collection reference guide. Version 1.0.0. ICHOM Diabetes in Adults Working Group, Type 1 and Type 2 Diabetes in Adults, November 2018. Available from https://ichom.org/files/medical-conditions/diabetes-in-adults/dia-reference-guide.pdf. Accessed 1 Dec 2019

[50] Carlton J, Peasgood T, Khan S, Barber R, Bostock J, Keetharuth AD (2020). An emerging framework for fully incorporating public involvement (PI) into patient-reported outcome measures (PROMs). J Patient Rep Outcomes.

