# Supplementary material for: The suitability of patient-reported outcome measures used to assess the impact of hypoglycaemia on quality of life in people with diabetes: a systematic review using COSMIN methods
Source: Diabetologia. 2021 Feb 2;64(6):1213–25. doi: 10.1007/s00125-021-05382-x (PMC8099839; doi:10.1007/s00125-021-05382-x)
Supplement: Supplementary file 1 — (PDF 281 KB) [file 125_2021_5382_MOESM1_ESM.pdf]

Electronic Supplementary Material

Methods

MEDLINE Search Strategy

Database: Ovid MEDLINE(R) and Epub Ahead of Print, In-Process & Other Non-Indexed Citations, Daily and Versions(R) <1946 to November 14, 2018>
Search Strategy:
----

1 exp Diabetes Mellitus, Type 1/ (71491)
2 (("typ* 1" or "typ* I") adj2 diabet*).tw. (45210)
3 (IDDM or T1DM or T1D).tw. (15691)
4 ("insulin* depend*" or "insulin depend*").tw. (29024)
5 exp Diabetes Mellitus, Type 2/ (117985)
6 ("non-insulin* depend*" or "noninsulin depend*").tw. (12172)
7 (("typ* 2" or "typ* II") adj2 diabet*).tw. (124595)
8 (NIDDM or T2DM or T2D).tw. (29798)
9 1 or 2 or 3 or 4 or 5 or 6 or 7 or 8 (242308)
10 exp Hypoglycemia/ (26705)
11 (hypoglycemi* or hypoglycaemi* or hypo-glycemi* or hypo-glycaemi* or low blood sugar or low blood glucose).mp. (102811)
12 10 or 11 (103401)
13 *Quality of Life/ or (patient reported outcome* or health-related quality of life or hrqol or hrql or health status or functional status or satisfaction or well-being or wellbeing or mood or depress* or distress or emotion* or self-efficacy or self-esteem or resilien* or fatigue or sleep or neuropsych* or cogniti* or worry or concern or “fear of hypoglyc*” or anxiety or anxious or burden or avoidance or blame or shame or stigma or economic* or financial or "work absence" or "absence from work" or productivity or absenteeism or presenteeism or cost analysis or quality adjusted life year* or qaly or disability adjusted life year* or daly).ti,ab. (1835728)
14 ((subjective or emotional) adj2 (wellbeing or well-being)).mp. (7163)
15 ((psychological or psychosocial or psycho-social) adj3 outcome*).mp. (7324)
16 ("physical health" or ((emotional or psychological) adj2 (well-being or wellbeing)) or "level of independence" or ((social or friend* or marital or partner* or husband* or wife* or spous* or family or familial or families) adj3 relationship*) or finances or (sleep* or fatigue* or tired* or vitality) or "daytime function*" or "cognitive function*" or attention or concentration or memory or ((fear or afraid or worr* or distress* or stigma* or impact*) adj3 (hypoglycaemi* or hypoglycemi*)) or anxi* or depress* or mood* or "psychological conflict").mp. (2846409)
17 ("care needs" adj3 (express* or perception* or perspective* or judge* or (patient* adj2 view*) or "own assessment*")).mp. (71)
18 13 or 14 or 15 or 16 or 17 (3965053)
19 instrumentation.fs. or Validation Studies.pt. or exp reproducibility of results/ or reproducib*.ti,ab. or exp psychometrics/ or psychometr*.ti,ab. or clinimetr*.ti,ab. or clinometr*.ti,ab. or exp observer variation/ or observer variation.ti,ab. or exp discriminant analysis/ or reliab*.ti,ab. or valid*.ti,ab. or coefficient.ti,ab. or internal consistency.ti,ab. or (cronbach* and (alpha or alphas)).ti,ab. or item correlation.ti,ab. or item correlations.ti,ab. or item selection.ti,ab. or item selections.ti,ab. or item reduction.ti,ab. or item reductions.ti,ab. or agreement.mp. or precision.mp. or imprecision.mp. or precise values.mp. or test-retest.ti,ab. or (test and retest).ti,ab. or (reliab* and (test or retest)).ti,ab. or stability.ti,ab. or interrater.ti,ab. or inter-rater.ti,ab. or intrarater.ti,ab. or intra-rater.ti,ab. or intertester.ti,ab. or inter-tester.ti,ab. or intratester.ti,ab. or intra-tester.ti,ab. or interobserver.ti,ab. or inter-observer.ti,ab. or intraobserver.ti,ab. or intra-observer.ti,ab. or intertechnician.ti,ab. or inter-

technician.ti,ab. or intratechnician.ti,ab. or intra-technician.ti,ab. or interexaminer.ti,ab. or inter-examiner.ti,ab. or intraexaminer.ti,ab. or intra-examiner.ti,ab. or interassay.ti,ab. or inter-assay.ti,ab. or intraassay.ti,ab. or intra-assay.ti,ab. or interindividual.ti,ab. or inter-individual.ti,ab. or intraindividual.ti,ab. or intra-individual.ti,ab. or interparticipant.ti,ab. or inter-participant.ti,ab. or intraparticipant.ti,ab. or intra-participant.ti,ab. or kappa.ti,ab. or kappa*.ti,ab. or coefficient of variation.ti,ab. or repeatab*.mp. or ((replicab* or repeated) and (measure or measures or findings or result or results or test or tests)).mp. or generaliza*.ti,ab. or generalisa*.ti,ab. or concordance.ti,ab. or (intraclass and correlation*).ti,ab. or discriminative.ti,ab. or known group.ti,ab. or factor analysis.ti,ab. or factor analyses.ti,ab. or factor structure.ti,ab. or factor structures.ti,ab. or dimensionality.ti,ab. or subscale*.ti,ab. or multitrait scaling analysis.ti,ab. or multitrait scaling analyses.ti,ab. or "item discriminant or interscale correlation".ti,ab. or interscale correlations.ti,ab. or ((error or errors) and (measure* or correlat* or evaluat* or accuracy or accurate or precision or mean)).ti,ab. or individual variability.ti,ab. or interval variability.ti,ab. or rate variability.ti,ab. or variability analysis.ti,ab. or (uncertainty and (measurement or measuring)).ti,ab. or standard error of measurement.ti,ab. or sensitiv*.ti,ab. or responsive*.ti,ab. or (limit and detection).ti,ab. or minimal detectable concentration.ti,ab. or interpretab*.ti,ab. or (small* and (real or detectable) and (change or difference)).ti,ab. or meaningful change.ti,ab. or minimal important change.ti,ab. or minimal important difference.ti,ab. or minimally important change.ti,ab. or minimally important difference.ti,ab. or minimal detectable change.ti,ab. or minimal detectable difference.ti,ab. or minimally detectable change.ti,ab. or minimally detectable difference.ti,ab. or minimal real change.ti,ab. or minimal real difference.ti,ab. or minimally real change.ti,ab. or minimally real difference.ti,ab. or ceiling effect.ti,ab. or floor effect.ti,ab. or Item response model.ti,ab. or IRT.ti,ab. or Rasch.ti,ab. or Differential item functioning.ti,ab. or DIF.ti,ab. or computer adaptive testing.ti,ab. or item bank.ti,ab. or cross-cultural equivalence.ti,ab. (4191101)

20 9 and 12 and 18 and 19 (1874)

Results

ESM Table 1 Quality assessment of this systematic review against COSMIN guidance

**COSMIN criteria**	**Review meets criteria**
Elements included in the research aim<br>Construct of interest<br>Population of interest<br>Type of measurement instrument of interest<br>Measurement properties of interest	 +<br>+<br>+<br>+
All available instruments included	+
Only instruments included that have at least some evidence of measurement properties	+
Search strategy described	+
No search terms or validated search filter used for:<br>Measurement properties<br>Type of instrument	 ±<br>-
Number of databases searched:<br>Search in at least 2 databases<br>Medline/PubMed<br>EMBASE<br>Additional databases<br>Reference checking used<br>No time limits used or good arguments for a times limit<br>No language restrictions used	 +<br>+<br>+<br>-<br>+<br>+<br>±
Inclusion and exclusion criteria clearly described	+
Reasons for excluding articles reported	+
Abstract selection by at least 2 reviewers?	+
Full-text article selection by at least 2 reviewers?	+
Abstract and full-text article selection by at least 2 reviewers?	±
Methodological quality of studies assessed	+
Quality assessment of studies done by at least 2 reviewers	+
Data on measurement properties extracted by at least 2 reviewers	+
Quality of the instrument (measurement properties) assessed	+
Quality assessment of the instrument by at least 2 reviewers	+
Results from multiple studies on the same instrument somehow combined (e.g. best evidence synthesis or pooling)	-
Data synthesis was performed:<br>Per measurement property<br>Only for domains (reliability, validity, responsiveness)<br>Only for the whole instrument	 +<br>-<br>-
Recommendation provided for the best instrument:<br>One instrument is recommended per construct<br>More instruments are recommended per construct<br>No recommendation for the best instrument	 +<br>-<br>-
Results for the measurement properties reported as raw data	+
Number of measurement properties reported	+
Conflict of interest or funding source declared	+
One of the authors of the review is also the developer of one of the instruments evaluated in the review	-

+ = criterion met; - = criterion not met; ± = criterion partly met

ESM Table 2 Summary of PROM construct definitions and COSMIN quality ratings

PROM	Reference	Original language	Author(s)' construct definition	Target population	Intended context of use	Concept elicitation study	
						COSMIN quality rating	Items generated by people with diabetes
FH-15	Anarte Ortiz et al, 2011 [39]	Spanish	"The consequences of hypoglycemia can be quite aversive and potentially life threatening. The physical sequelae provide ample reason for patients to fear hypoglycemia and avoid episodes"	T1DM	Clinical care and research	Very good	No
HFS	Cox et al, 1987 [32]	English (US)	"Fear of hypoglycemia (…) a significant psychological barrier to diabetic adherence. (…) Excessive fear of hypoglycemia may have clinical significance as a cause of poor adherence"	Adults with diabetes (type not reported)	Not explicitly stated	Inadequate	Yes
HFS-II	Gonder-Frederick et al, 2011 [40]	English (US)	Assumed the same as HFS	Assumed the same as HFS	Not explicitly stated	Doubtful	Unclear
HABS	Polonsky et al 2015 [42]	English (US)	"Worries and concerns about hypoglycemia among adults with diabetes have a deleterious impact on glycemic control and quality of life (…) there may be important differences in the experience of hypoglycemic worries and concerns between those with T1D and those with T2D given the relatively limited exposure to hypoglycemia in T2Ds compared to T1Ds"	T2DM	Clinical care	Inadequate	Yes
HCS	Polonksy et al 2017 [43]	English (US)	"hypoglycemic confidence encompasses a sense of personal strength and comfort derived from the belief that one has the necessary resources to stage safe from hypoglycemia-related problems; it can therefore be viewed as	T1DM, T2DM	Research and clinical care	Doubtful	Yes

PROM	Reference	Original language	Author(s)' construct definition	Target population	Intended context of use	Concept elicitation study	
						COSMIN quality rating	Items generated by people with diabetes
			representing the positive side of hypoglycemic fear and avoidance"				
QoLHYPO	Orozco-Beltrán et al, 2018 [33]	Spanish	"hypoglycemia is a major barrier to achieving the glycemic goal in T2DM patients, and the impact on HRQoL and worry they cause can lead to the patient developing self-regulation attitudes towards medication and low adherence to it to avoid them, incorporating the examination of the impact of hypoglycemia on HRQoL"	T2DM	Clinical care	Inadequate	Yes
TRIM-HYPO	Brod et al, 2015 [34]	English (US)	"Symptoms [of non-severe hypoglycemic events] can have a serious impact on a person's quality of life, health, psychological well-being, and adherence to treatment regimens..."	T1DM, T2DM	Research and clinical care	Doubtful	Yes

T1DM = type 1 diabetes; T2DM = type 2 diabetes

ESM Table 3 Characteristics of development studies of included PROMs

PROM	Reference	Population characteristics			Diabetes characteristics		Instrument administration			Response rate
		N	Age Mean (SD, range) years	Gender % female	Diabetes type	Diabetes duration mean (SD) years	Setting/ Mode of administration	Country	Language	
FH-15	Anarte Ortiz et al, 2011 [39]	Survey: 229	34.6 (11.0)	57.6	T1DM	16.1 (10.1)	Clinic; presumed paper	Spain	Spanish	NR
HFS	Cox et al, 1987 [32]	Item generation: 20 interviews with PwD (and HCP, n=NR)<br><br>Pilot: 12 PwD (and HCP, n=NR)	NR<br><br>NR	NR<br><br>NR	Not explicitly stated DM type, “insulin-requiring”.	NR<br><br>NR	Clinic; presumed paper	USA	English (USA)	NR
HFS-II	Gonder-Frederick et al, 2011 [40]	NR	NR	NR	Presumed as per HFS	NR	Clinic; presumed paper	USA	English (USA)	NR
HABS	Polonsky et al, 2015 [42]	Item generation: 16 interviews PwD (and 11 HCPs)	NR	NR	T2DM	NR	Clinic; presumed paper	USA	English (USA)	NR
		Survey: 424	58.1 (11.4)	273 (64.4)		11.6 (9.2)				

PROM	Reference	Population characteristics			Diabetes characteristics		Instrument administration			Response rate
		N	Age Mean (SD, range) years	Gender % female	Diabetes type	Diabetes duration mean (SD) years	Setting/ Mode of administration	Country	Language	
HCS	Polonksy et al, 2017 [43]	Item generation: 12 interviews PwD (and HCP, n=7)<br><br>Survey: 553	NR<br><br>T1DM 48.7 (14.8) T2DM (basal and prandial insulin) 60.1 (12.3) T2DM basal insulin 59.0 (11.1)	NR<br><br>T1DM 232 (71.2) T2DM (basal and prandial insulin) 93 (64.1) T2DM basal insulin 49 (59.8)	T1DM (n=6), T2DM (n=6)<br><br>T1DM (n=326) T2DM (n=227)	NR<br><br>T1DM 25.8 (14.9) T2DM (basal and prandial insulin) 17.6 (9.3) T2DM basal insulin 14.4 (9.6)	Clinic; presumed paper	USA	English (USA)	NR
QoLHYPO	Orozco-Beltrán et al, 2018 [33]	Item generation: 10 interviews PwD (and 2 focus groups HCP, n=4)	NR	NR	T2DM	NR	Clinic; presumed paper	Spain	Spanish	NR

PROM	Reference	Population characteristics			Diabetes characteristics		Instrument administration			Response rate
		N	Age Mean (SD, range) years	Gender % female	Diabetes type	Diabetes duration mean (SD) years	Setting/ Mode of administration	Country	Language	
		Face validity: 18 PwD<br><br>Pilot test: 140 PwD	NR<br><br>NR	NR<br><br>NR	T2DM<br><br>T2DM	NR<br><br>NR				
TRIM-HYPO	Brod et al, 2015 [34]	Item generation: 146 PwD<br><br>Cognitive interviews: 21<br><br>Survey: 407	Total: 44.4 (NR) T1DM 38.1 (NR) T2DM 48.4 (NR)<br><br>NR<br><br>50.2 (18.0)	Total: 72 (49.3) T1DM 26 (45.6) T2DM 46 (51.7)<br><br>NR<br><br>200 (49%)	T1DM 57 T2DM 89<br><br>NR<br><br>T1DM 133 (32.7) T2DM 274 (67.3)	Total: 12.6 (NR) T1DM 19.3 (NR) T2DM 8.2 (NR)<br><br>NR<br><br>NR	Not clear; presumed online and paper-based	France, Germany, UK, USA	English (US)	NR

HCP = health care professionals; NR = not reported; T1DM = type 1 diabetes; T2DM = type 2 diabetes

ESM Table 4 Consensus reviewer ratings of PROMs

PRO		Clinicians (n=6)	Researchers (n=6)	People with diabetes (n=4)	Consensus
**FH-15**	RELEVANCE RATING (+ / - / ± / ?)	+	+	+	**+**
	COMPREHENSIVENESS RATING (+ / - / ± / ?)	+	-	+	**+**
	COMPREHENSIBILITY RATING (+ / - / ± / ?)	+	+	?	**+**
	**CONTENT VALIDITY RATING (+ / - / ± / ?)**	**+**	**±**	**+†**	**+**
**HFS**	RELEVANCE RATING (+ / - / ± / ?)	+	±	+	**+**
	COMPREHENSIVENESS RATING (+ / - / ± / ?)	±	-	+	**±**
	COMPREHENSIBILITY RATING (+ / - / ± / ?)	+	+	+	**+**
	**CONTENT VALIDITY RATING (+ / - / ± / ?)**	**+†**	**±**	**+**	**+†**
**HFS-II**	RELEVANCE RATING (+ / - / ± / ?)	+	±	+	**+**
	COMPREHENSIVENESS RATING (+ / - / ± / ?)	-	-	+	**-**
	COMPREHENSIBILITY RATING (+ / - / ± / ?)	+	+	+	**+**
	**CONTENT VALIDITY RATING (+ / - / ± / ?)**	**±**	**±**	**+**	**±**
**HABS**	RELEVANCE RATING (+ / - / ± / ?)	+	+	+	**+**
	COMPREHENSIVENESS RATING (+ / - / ± / ?)	-	-	+	**-**
	COMPREHENSIBILITY RATING (+ / - / ± / ?)	+	+	+	**+**
	**CONTENT VALIDITY RATING (+ / - / ± / ?)**	**±**	**±**	**+**	**±**
**HCS**	RELEVANCE RATING (+ / - / ± / ?)	±	±	+	**±**
	COMPREHENSIVENESS RATING (+ / - / ± / ?)	-	-	+	**-**
	COMPREHENSIBILITY RATING (+ / - / ± / ?)	+	+	+	**+**
	**CONTENT VALIDITY RATING (+ / - / ± / ?)**	**±**	**±**	**+**	**±**
**QoLHYPO**	RELEVANCE RATING (+ / - / ± / ?)	±	+	+	**+**
	COMPREHENSIVENESS RATING (+ / - / ± / ?)	±	+	+	**+**

	COMPREHENSIBILITY RATING (+ / - / ± / ?)	±	±	+	±
	**CONTENT VALIDITY RATING (+ / - / ± / ?)**	**±**	**+†**	**+**	**+†**
**TRIM-HYPO**	RELEVANCE RATING (+ / - / ± / ?)	+	+	±	+
	COMPREHENSIVENESS RATING (+ / - / ± / ?)	-	-	±	-
	COMPREHENSIBILITY RATING (+ / - / ± / ?)	+	+†	+	+
	**CONTENT VALIDITY RATING (+ / - / ± / ?)**	**±**	**±**	**±†**	**±**

+ sufficient; - inadequate; ± inconsistent; ? indeterminate

† denotes no COSMIN guidance on ratification

ESM Table 5 Characteristics, assessment, and results of structural validity* studies for included PROMs

PROM	Reference	Country (language)	Study participants' characteristics			PROM score	COSMIN quality rating	Analysis model	Results (synthesis)
			N	Age Year, month (SD)	Duration of diabetes Year, month (SD)				
FH-15	Anarte Ortiz et al, 2011 [39]	Spain (Spanish)	229	34.6 (18.5)	16.1 (10.1)	NR	Adequate	Exploratory	Initially employed principal-components method and promax rotation which gave 5-factor solution. Items from the final two factors were removed due to low correlations (<0.4), then undertook EFA on remaining scale. EFA showed scale to be made up of three factors that accounted for 58.27% of the common variance of the 15 items analysed.
HFS	Cox et al, 1987 [32]	USA (English)	158	38.1 (16.7)	12 (8.6)	64 ± 17	Adequate	Exploratory	Varimax rotation revealed one factor that loaded on the worry subscale. It accounted for 40% of variance
HFS-II	Gonder-Frederick et al, 2011 [40]	USA (English)	289	(aggregate sample) 41.9 (12.6)	(aggregate sample) 23.8 (12.5)	Study 1 52.2 (24.8); study 2 43.5 (20.6); study 3 39.3 (18.7)	Adequate	Exploratory	Fit estimates for a one-factor solution questionably acceptable X^2 (79) = 1,288.4, p<0.0001; RMSEA = 0.14, SRMR = 0.13, TLI = 0.92.Two-factor solution improved fit X^2 (102) = 973.8, p<0.0001; RMSEA = 0.10, SRMR = 0.08, TLI = 0.95. A three X^2factor

PROM	Reference	Country (language)	Study participants' characteristics			PROM score	COSMIN quality rating	Analysis model	Results (synthesis)
			N	Age Year, month (SD)	Duration of diabetes Year, month (SD)				
									model was tested and fit the data (112) = 630.1, p<0.0001; RMSEA = 0.08, SRMR = 0.06, TLI = 0.98.
HFS Norwegian	Graue et al, 2013 [36]	Norway (Norwegian)	235	39.4 ± 13.7	NR	NR	Very good	Exploratory	EFA yielded a 7-factor solution, yet scree plot indicated 4-factor solution. EFA showed CFI=0.87, TLI=0.85, RMSEA=0.066. For CFA to 2-factors (as per developers), Fit indices were CFI=0.75, TLI=0.72, RMSEA=0.088. 4-factor solution had better fit: CFI = 0.87, TLI = 0.85 and RMSEA = 0.066.<br>CFA showed significant positive factor loadings. HFS-B 0.94-2.40, HFS-W 0.83-1.65. Fit indices were CFI = 0.75, TLI = 0.72 and RMSEA = 0.088
HFS Singapore	Lam et al, 2017 [38]	Singapore (Mandarin)	144	45.9 (14.2)	14.8 (8.7)	24.40 (17.92)	Very good	Confirmatory	CFA yielded model fit RMSEA =0.094, CFI=0.777, TLI=0.762, SRMR=0.081 suggesting poor fit between the 2-factor structure in the original and current data. Subsequent EFA with varimax rotation

PROM	Reference	Country (language)	Study participants' characteristics			PROM score	COSMIN quality rating	Analysis model	Results (synthesis)
			N	Age Year, month (SD)	Duration of diabetes Year, month (SD)				
									extracted seven factors. Weighing the increase in total variance explained and the interpretability of the factors, authors concluded that the 3-factor solution is the best model
HFS Spanish	Tasende et al, 2018 [35]	Spain (Spanish)	163	36 (10.5)	17.7 (9.7)	NR	Adequate	Exploratory	Factor analysis identified 3 components. Subscale 1 (worry) accounted for 42% of the global variance, subscale 2 (behaviour associated with the avoidance) accounted for 6.5%, and subscale 3 (behavior causing hypoglycemia)
HFS Swedish	Anderbro et al, 2008 [35]	Sweden (Swedish)	322	47.7 (14.7)	24.0 (13.0)	NR	Adequate	Principal components with varimax rotation	Factor analysis indicated a scale consisting of three factors: factor 1 (worry) eigenvalue 6.4 which accounted for 28% of response variance; factor 2 (avoidance) eigenvalue 2.3, which accounted for 10% response variance; factor 3 (aloneness) eigenvalue 1.5 accounted for 6.4% response variance

PROM	Reference	Country (language)	Study participants' characteristics			PROM score	COSMIN quality rating	Analysis model	Results (synthesis)
			N	Age Year, month (SD)	Duration of diabetes Year, month (SD)				
HFS-II short-form	Grabman et al, 2016 [31]	USA, Turkey, Slovenia (English)	Dataset 1: 487 Dataset 2: 2718	Dataset 1: 43.9 (3.9) Dataset 2: 58.7 (15.8)	Dataset 1: 17.9 (11.7) Dataset 2: 19.0 (11.2)	NR	Very good	Exploratory	EFA on dataset 2 with direct oblimin rotation. A 3-factor solution was indicated (worry, avoidance and maintain high). When sample split by T1DM and T2DM, EFA for T1DM suggested 3-factor solutions for both
HABS	Polonsky et al, 2015 [42]	USA (English)	424	58.1 (11.4)	11.6 (9.2)	NR	Adequate	Exploratory	EFA of the original 30 items yielded a 4-factor solution for each sample that accounted for >55% of the common item variance. Once items were dropped, final EFA yielded three factors in each sample that accounted for >55.6% in the non-insulin group and >61.3% in the insulin group
HCS	Polonsky et al, 2017 [43]	USA (English)	553	T1DM 48.7 (14.8) T2DM-BP 60.1 (12.3) T2DM-BO 59.0 (11.1)	T1DM 25.8 ± 14.9 T2DM-BP 17.6 ± 9.3 T2DM-BO 14.4 (9.6)	NR	Adequate	Exploratory	EFAs yielded a single factor solution for each of the samples accounting for 50.8%, 65.1% and 73.7% respectively. All factor loadings for all three analysis were ≥0.50 and ranged from 0.55 to 0.92

PROM	Reference	Country (language)	Study participants' characteristics			PROM score	COSMIN quality rating	Analysis model	Results (synthesis)
			N	Age Year, month (SD)	Duration of diabetes Year, month (SD)				
QoLHYPO	Orozco-Beltrán et al, 2018 [33]	Spain (Spanish)	227	62.7 (11.0)	12.6 (7.4)	NR	Adequate	Exploratory	Results showed consisted of a single factor and was therefore unidimensional
TRIM-HYPO	Brod et al, 2015 [34]	USA English (US)	407	50.2 (18.0)	NR	Not explicitly reported	Adequate	Exploratory	Principal component solutions were conducted for each hypothesized domain (five independent models) with the extraction based on one fixed number of factors. These models explained 64.9–72.1 % of total variances. The principal component analyses (PCA) supported the preliminary theoretical framework based on the TRIM-HYPO having five distinct domains: daily function, emotional well-being, diabetes management, sleep disruption, and work productivity.

*Structural validity definition: The extent to which the items in a PROM reflect the dimensionality of the construct (i.e. the items form a single (unidimensional) scale or multiple sub-scales (a multidimensional scale)

CFI = comparative fit index; EFA = exploratory factor analysis; RMSEA = root mean square error of approximation; TLI = Tucker Lewis index; T2DM-BP = basal and prandial insulin group; T2DM-BO = basal only insulin group

ESM Table 6 Characteristics of studies in which internal consistency reliability* was assessed

PROM	Reference	Country (language)	Study participants' characteristics			PROM score	COSMIN quality rating	Statistical method	Results (synthesis)
			N	Age Year, month (SD)	Duration of diabetes Year, month (SD)				
FH-15	Anarte Ortiz et al, 2011 [39]	Spain (Spanish)	229	34.6 (18.5)	16.11 (10.1)	NR	Adequate	Cronbach's α	Total FH-15 0.891; subscales all had alpha values greater than 0.75
HFS	Cox et al, 1987 [32]	USA (English)	158	38.1 (16.7)	12 (8.6)	64 ± 17	Adequate	Cronbach's α	Total HFS 0.90; behavior subscale 0.60; worry subscale 0.89
HFS-II	Gonder-Frederick et al, 2011 [40]	USA (English)	289	(aggregate sample) 41.9 (12.6)	(aggregate sample) 23.8 (12.5)	Study 1 52.2 (24.8); study 2 43.5 (20.6); study 3 39.3 (18.7)	Adequate	Cronbach's α	Total HFS-II 0.94; behavior subscale 0.85; worry subscale 0.94
HFS-II	Pinhas-Hamiel et al, 2017 [41]	Israel (English)	53	27.8 (8.2)	14.0 (8.0)	65.7 ± 16.5	Very good	Cronbach's α	Internal consistency of 0.90. Behaviour subscale 0.79; worry subscale 0.89
HFS Norwegian	Graue et al, 2013 [37]	Norway (Norwegian)	235	39.4 (13.7)	NR	NR	Very good	Cronbach's α	HFS-II total 0.90; HFS-B 0.92; HFS-W 0.87
HFS Singapore	Lam et al, 2017 [38]	Singapore (Mandarin)	144	45.9 (14.2)	14.8 (8.7)	24.40 (17.92)	Very good	Cronbach's α	Total HFS 0.93; behavior subscale 0.84; worry subscale 0.95

PROM	Reference	Country (language)	Study participants' characteristics			PROM score	COSMIN quality rating	Statistical method	Results (synthesis)
			N	Age Year, month (SD)	Duration of diabetes Year, month (SD)				
HFS Spanish	Tasende et al, 2018 [36]	Spain (Spanish)	163	36 (10.5)	17.7 (9.7)	NR	Adequate	Cronbach's α	Worry subscale 0.939; behavior associated with avoidance subscale 0.6; behavior causing hypoglycemia subscale 0.728
HFS-II short-form	Grabman et al, 2017 [31]	USA, Turkey, Slovenia (English)	Dataset 1: 487 Dataset 2: 2718	Dataset 1: 43.9 (3.9) Dataset 2: 58.7 (15.8)	Dataset 1: 17.9 (11.7) Dataset 2: 19.0 (11.2)	NR	Very good	Cronbach's α	Avoidance subscale 0.76; maintain high subscale 0.84; worry subscale 0.82; total scale 0.84
HABS	Polonsky et al, 2015 [42]	USA (English)	424	58.1 (11.4)	11.6 (9.2)	NR	Adequate	NR	Hypoglycemia anxiety: α 0.85 for insulin users, 0.83 for non-insulin users; hypoglycemia avoidance α 0.77 insulin users, 0.74 non-insulin users; hypoglycemia confidence α 0.80 insulin users, 0.73 non-insulin users

PROM	Reference	Country (language)	Study participants' characteristics			PROM score	COSMIN quality rating	Statistical method	Results (synthesis)
			N	Age Year, month (SD)	Duration of diabetes Year, month (SD)				
HCS	Polonsky et al, 2017 [43]	USA (English)	553	T1DM 48.7 (14.8) T2DM-BP 60.1 (12.3) T2DM-BO 59.0 (11.1)	T1DM 25.8 (14.9) T2DM-BP 17.6 (9.3) T2DM-BO 14.4 (9.6)	NR	Adequate	Cronbach's α	T1DM 0.87; T2DM-BP 0.93; T2DM-BO 0.95
QoLHYPO	Orozco-Beltrán et al, 2018 [33]	Spain (Spanish)	227	62.7 (11.0)	12.6 (7.4)	NR	Adequate	Cronbach's α	Visit 1 0.912; visit 2 0.901
TRIM-HYPO	Brod et al, 2015 [34]	USA (English)	407	50.2 (18.0)	NR	NR	Very good	Cronbach's α	Daily function subscale (DF, 7 Items) 0.909; Emotional well-being subscale (EWB, 7 items) 0.918; Diabetes management subscale (DM, 5 items) 0.897; Sleep disruption subscale (SD, 5 items) 0.862; Work productivity subscale (WP, 9 items) Work productivity subscale (WP, 9 items) 0.951; TOTAL 3 domains:

									DF, EWB, DM (total, 19 items) 0.954

NR = not reported; T1DM = type 1 diabetes; T2DM = type 2 diabetes

* Internal consistency definition: The extent to which there is consistency of results across items in the PROM (i.e., within a specified scale or subscale)

ESM Table 7 Characteristics of studies in which test-retest reliability* was assessed

PROM	Reference	Country (language)	Study participants' characteristics			PROM score	COSMIN quality rating	Analysis model	Results (synthesis)
			N	Age Year, month (SD)	Duration of diabetes Year, month (SD)				
HFS	Cox et al, 1987 [32]	USA (English)	Set 1: 22, set 2: 22	Set 1: 32.4 Set 2: 44.3 (7.9)	Set 1: 7.8 Set 2: 25.7 (8.5)	Total pretest: 60.0 ± 13.8 Total post-test 62.3 ± 7.9	Adequate	NR	Set 1: 0.68 p<0.018 total scale; 0.68 p<0.009 behaviour subscale; 0.64 p<0.013 worry subscale<br>Set 2: 0.89 p<0.01 total scale; 0.81 p<0.001 behaviour subscale; 0.85 p<0.001 worry subscale
HFS-II	Gonder-Frederick et al, 2011 [40]	USA (English)	Study 3 113	NR	NR	study 3 39.3 (18.7)	Adequate	Repeated baseline design, temporal reliability	Total HFS-II Cronbach's α 0.74; behavior subscale 0.81; worry subscale 0.63
HFS Norwegian	Graue et al, 2013 [37]	Norway (Norwegian)	235	39.4 (13.7)	NR	NR	Very good	ICC	ICCs were 0.77 for HFS-B, 0.84 for HFS-W and 0.82 for HFS-II total score
HFS Singapore	Lam et al, 2017 [38]	Singapore (Mandarin)	22 (test-retest group)	NR	NR	NR	Very good	ICC	Total HFS-II 0.751; HFS-B 0.627; HFS-W 0.723
HFS Spanish	Tasende et al, 2018 [36]	Spain (Spanish)	163	36 (10.5)	17.7 (9.7)	NR	Adequate	Test-retest correlation	Global EsHFS r=0.92 (95% CI 0.84-0.96)

									p<0.001; worry subscale r=0.91 (95% CI 0.82-0.96) p<0.001; avoidance subscale r=0.46 (95% CI 0.13-0.70) p=0.009; hyperglycemia causing behavior subscale r=0.91 (95% CI 0.82-0.96) p<0.001
QoLHYPO	Orozco-Beltrán et al, 2018 [33]	Spain (Spanish)	142 in reliability cohort	NR for reliability cohort	NR for reliability cohort	NR	Adequate	Test-retest reliability using ICC for the total score and Cohen's kappa coefficient for the responses to each item	Good reliability (ICC = 0.920 (CI 95% 0.890-0.942) and kappa >0.60 for all items
TRIM-HYPO	Brod et al, 2015 [34]	USA (English)	407	50.2 (18.0)	NR	NR	Doubtful	Test–retest reliability was assessed using ICC in a subsample, within 3 weeks after completion	Daily function subscale (DF, 7 Items) 0.746; Emotional well-being subscale (EWB, 7 items) 0.885; Diabetes management subscale (DM, 5 items) 0.791; Sleep

								of the initial battery	disruption subscale (SD, 5 items) 0.982; Work productivity subscale (WP, 9 items) Work productivity subscale (WP, 9 items) 0.870; TOTAL 3 domains: DF, EWB, DM (total, 19 items) 0.836

ICC = intraclass correlation coefficient; NR = not reported;

* Reliability test-retest definition: The extent to which the PROM yields scores that are reproducible (stable) over time when there has been no change in the concept being assessed

ESM Table 8 Characteristics of studies in which hypotheses testing for construct validity was assessed

PROM	Reference	Country (language)	Study participants' characteristics			PROM score	COSMIN quality rating	Analysis model	Results (synthesis)
			N	Age Year, month (SD)	Duration of diabetes Year, month (SD)				
FH-15	Anarte Ortiz et al, 2011 [39]	Spain (Spanish)	229	34.6 (18.5)	16.1 (10.1)	NR	Adequate	Student's t-test	Significant difference between participants who had FH and those who did not t(171.530) = 10.975, p<0.001, with higher scores for those with FH.
HFS-II	Gonder-Frederick et al, 2011 [40]	USA (English)	289	(aggregate sample) 41.9 (12.6)	(aggregate sample) 23.8 (12.5)	Study 1 52.2 (24.8); study 2 43.5 (20.6); study 3 39.3 (18.7)	Adequate	Correlations for convergent validity	HFS-B and HFS-W subscales significantly correlate with Modified State-Trait Personality Inventory Anxiety, Anger and Depression subscales. Both also significantly correlate with SF-12 Physical and Mental scores. Discriminant validity: Significant differences for

PROM	Reference	Country (language)	Study participants' characteristics			PROM score	COSMIN quality rating	Analysis model	Results (synthesis)
			N	Age Year, month (SD)	Duration of diabetes Year, month (SD)				
									experience of severe hypoglycaemia F (1,281)=8.956, p=0.003 (HFS-B) and F (1,770)=63.037, p<0.0005 (HFS-W), effect sizes 0.35 (HFS-B) and 0.58 (HFS-W)
HFS Singapore	Lam et al, 2017 [38]	Singapore (Mandarin)	144	45.9 (14.2)	14.8 (8.7)	24.40 (17.92)	Very good	Correlation coefficients for convergent validity;	A weak positive correlation between HFS-B scores and the PHQ-9 and GAD-7 scores (0.202 and 0.241), and a moderate positive correlation between HFS-II and HFS-W and the PHQ-9 and GAD-7 scores (0.395 and 0.432).<br>HFS-II and HFS-W scores were significantly higher for those with severe hypoglycemia in the past year than those

PROM	Reference	Country (language)	Study participants' characteristics			PROM score	COSMIN quality rating	Analysis model	Results (synthesis)
			N	Age Year, month (SD)	Duration of diabetes Year, month (SD)				
									without (F (1,142)=7.45 p=0.007; (F (1,142)=7.17, p=0.008) with medium effect sizes of 0.49 for HFS-II and 0.47 for HFS-W. No significant difference for HFS-B.
HFS Spanish	Tasende et al, 2018 [36]	Spain (Spanish)	163	36 (10.5)	17.7 (9.7)	NR	Adequate	Pearson correlation coefficients	Correlated to Spanish version of DQOL (0.424, p<0.001)
HFS Swedish	Anderbro et al, 2008 [35]	Sweden (Swedish)	322	47.7 (14.7)	24.0 (13.0)	Total score mean 25.0 (10.8)	Adequate	Correlation coefficients	Swe-HFS and SWE-PAID-20, total Swe-HFS correlated positively with total PAID (r=0.44, p=0.01)
HFS-II short-form	Grabman et al, 2017 [31]	USA, Turkey, Slovenia (English)	Dataset 1: 487 Dataset 2: 2718	Dataset 1: 43.9 (3.9) Dataset 2: 58.7 (15.8)	Dataset 1: 17.9 (11.7) Dataset 2: 19.0 (11.2)	NR	Very good	Correlation coefficients for convergent validity	Scale means on the short-form and long form showed strong positive correlations for the avoidance subscale (r=0.89), p<0.0005; maintain high subscale

PROM	Reference	Country (language)	Study participants' characteristics			PROM score	COSMIN quality rating	Analysis model	Results (synthesis)
			N	Age Year, month (SD)	Duration of diabetes Year, month (SD)				
									(r=0.91, p<0.0005); worry subscale (r=0.94, p<0.0005) and total scale (r=0.95, p<0.0005)
HABS	Polonsky et al, 2015 [42]	USA (English)	424	58.1 (11.4)	11.6 (9.2)	NR	Adequate	Correlation coefficients for convergent validity	Higher anxiety and Avoidance scores were significantly associated with: lower well-being, greater diabetes distress, more symptoms of depression and anxiety all p<.05. Similar significant associations found in the converse direction for Confidence Scale. Higher A1C levels were significantly associated with greater Avoidance and lower Confidence, but only for insulin users

PROM	Reference	Country (language)	Study participants' characteristics			PROM score	COSMIN quality rating	Analysis model	Results (synthesis)
			N	Age Year, month (SD)	Duration of diabetes Year, month (SD)				
									versus non-insulin users
HCS	Polonsky et al, 2017 [43]	USA (English)	553	T1DM 48.7 (14.8)<br>T2DM-BP 60.1 (12.3)<br>T2DM-BO 59.0 (11.1)	T1DM 25.8 (14.9)<br>T2DM-BP 17.6 (9.3)<br>T2DM-BO 14.4 (9.6)	NR	Adequate	Correlation coefficients	Convergent validity: HCS scores were significantly and negatively linked with global anxiety and positively associated with well-being and lower A1C, but only for T1D and T2D-BP. Discriminant validity: greater years of education were associated with greater hypoglycemic confidence for all three samples (r=0.15 for T1D, r=0.21 T2-BP, r=0.30 T2-BO, p<0.05)
QoLHYPO	Orozco-Beltrán et al, 2018 [33]	Spain (Spanish)	227	62.7 (11.0)	12.6 (7.4)	Not reported	Adequate	Spearmans' correlation coefficient	Significant correlation (rho=0.557), p<0.001 between QoLHYPO and the

PROM	Reference	Country (language)	Study participants' characteristics			PROM score	COSMIN quality rating	Analysis model	Results (synthesis)
			N	Age Year, month (SD)	Duration of diabetes Year, month (SD)				
									ADDQoL=19. Significant correlation between QoLHYPO and EQ-5D-3L (mobility rho =-0.216, p0.001); self-care (rho=-0.259 p<0.01) usual activities (rho =-0.032 p<0.01); pain/discomfort (rho=0.265 p<0.01); anxiety/depression (rho =-0.346 p<0.01, VAS (rho =-0.446, p<0.01)
TRIM-HYPO	Brod et al, 2015 [34]	USA (English)	407	50.2 (18.0)	NR	NR	Adequate	Pearson's correlation coefficients	Convergent validity: Daily function and Activity Impairment Assessment (AIA) total score r = 0.473*; Emotional well-being and Psychological General Well-Being Index (PGWBI) global Score r = -0.467*; Diabetes

									management and Insulin Treatment Satisfaction Questionnaire (ITSQ)-HYPO Control Domain Score r = 0.551*; Sleep disruption and Medical Outcomes Sleep (MOS-Sleep)—problems index I r = 0.432*; Medical Outcomes Sleep (MOS-Sleep)—problems index II r = 0.453*; Work productivity and Work Productivity and Activity Impairment (WPAI)—percent overall work impairment due to problem r = 0.543*; Total 3 domain and Sheehan Disability Scale (SDS) r = 0.545*<br>Where *is significant at the 0.01 level (2-tailed)<br>Discriminant validity:

									Able to discriminate between expected known relationships, and all but one of the known-groups hypotheses were confirmed

NR = not reported; T1DM = type 1 diabetes; T2DM = type 2 diabetes

* Construct validity definition: The extent to which the scores of a PROM are consistent with hypotheses. For example, with regard to internal relationships, relationships to scores of other instruments, or differences between relevant groups. It is based on the assumption that the PROM is a valid measure of the construct
